# High Energy electron and proton acceleration by circularly polarized laser pulse from near critical density hydrogen gas target

**DOI:** 10.1038/s41598-018-20506-x

**Published:** 2018-02-01

**Authors:** Ashutosh Sharma

**Affiliations:** ELI-ALPS, ELI-HU Non-Profit Ltd., Dugonics ter 13, H-6720 Szeged, Hungary

## Abstract

Relativistic electron rings hold the possibility of very high accelerating rates, and hopefully a relatively cheap and compact accelerator/collimator for ultrahigh energy proton source. In this work, we investigate the generation of helical shaped quasi-monoenergetic relativistic electron beam and high-energy proton beam from near critical density plasmas driven by petawatt-circularly polarized-short laser pulses. We numerically observe the efficient proton acceleration from magnetic vortex acceleration mechanism by using the three dimensional particle-in-cell simulations; proton beam with peak energy 350 MeV, charge ~10nC and conversion efficiency more than 6% (which implies 2.4 J proton beam out of the 40 J incident laser energy) is reported. We detailed the microphysics involved in the ion acceleration mechanism, which requires investigating the role of self-generated plasma electric and magnetic fields. The concept of efficient generation of quasi-monoenergetic electron and proton beam from near critical density gas targets may be verified experimentally at advanced high power – high repetition rate laser facilities e.g. ELI-ALPS. Such study should be an important step towards the development of high quality electron and proton beam.

## Introduction

Ion acceleration with high-intensity lasers has attracted a great deal of attention because accelerated ion beams have extreme laminarity, ultrashort duration and high particle number in MeV energy range. These phenomenal characteristics of ion beam prompted excogitation about a wide range of applications in nuclear and medicine physics^1–3^. The requirement of ion beam with narrow energy spread, high conversion efficiency and compatibility of target with the high-repetition rate laser system, still a challenging task despite a decade plus efforts^4^.

The widely employed schemes for laser driven ion acceleration include target normal sheath acceleration (TNSA)^5–7^, radiation pressure acceleration (RPA)^8–16^, breakout afterburner (BOA)^17^, collisionless shockwave acceleration (CSA)^18–20^ and magnetic vortex acceleration (MVA)^21–24^. The most stable and well-understood mechanism so far is the TNSA^5–7^, which usually requires long pulse duration in order to reach high cut-off energy and thin solid target which allows us to get very sharp density gradients and high accelerating fields. At high repetition rate, using such targets raises significant challenges with debris, target insertion and unwanted secondary radiation such as bremsstrahlung. In comparison to typical ion acceleration experiments which utilizes a laser-thin solid foil interaction; MVA in near critical density (NCD) plasmas may be realized in a high density gas jet^25^ which are considered to have an advantage of higher laser-plasma coupling. The use of NCD target allows the MVA mechanism to generate high-energy ions at high repetition from a high purity proton source, which is attractive for applications required high repetition rate with the solid-state lasers. Recently MVA mechanism, employing the NCD target interaction with the linearly polarized (LP) laser pulses has been investigated theoretically^21–24^ and experimentally^26,27^ and attracted a great deal of attention due to its prediction for achieving sub-relativistic to relativistic electron and proton source.

It has been recognized that gas jet targets offer the possibility for high repetition rates^28,29^, however their performance as proton sources has been limited to low particle energies and yields^26^. The highest proton beam quality has been observed when applying solid-density planar target geometries^30^; so far the highest proton energies experimentally achieved with TNSA are 85 MeV^31^. In our previous research^23^, we demonstrated the proton acceleration via MVA mechanism with tightly focused LP laser pulse (2PW-20fs) interaction with NCD plasma (~3.0 n_c_). Simulation results in this regime shows comparatively lower laser-to-proton conversion efficiency ~1% (for protons with energy >4 MeV) but higher peak proton energy. However, medical applications require quasi-monoenergetic ion beams at hundreds of MeV with efficiency ~10%. For the detailed review of laser-driven ion acceleration see refs^4,30^.

In this work, we focused on the aspect of efficient proton acceleration from MVA mechanism where we have used the circularly polarized (CP) laser pulse interaction with NCD plasma target. So far, there is less attention paid to CP laser driven MVA mechanism and subsequently on efficient generation of electron and proton beam, which is needed, for applications. However intense CP laser interaction with plasma channels has shown great potential for the generation of megagauss axial magnetic field^32^ which has both fundamental and application interest such as particle acceleration and collimation. The preference of CP over LP laser pulse enabled the generation of axial and azimuthal magnetic fields in plasma channel. This new insight of polarization dependence in MVA mechanism of ion acceleration, has resulted the generation of quasi-monoenergetic helical electron beam and high energy quasi-monoenergetic proton beam with high proton yields.

MVA mechanism^22^ of ion acceleration can be realized when the laser pulse propagates through a NCD target that is much longer than the laser pulse itself. As tightly focused laser pulse interacts with the target, the ponderomotive force of the laser pulse drives electron cavitation. Plasma electrons become trapped and accelerated within these cavitation. These fast electrons trail behind the laser front, forming an axial fast current. A cold electron return current is formed to balance the fast current to maintain the plasma quasi-neutrality. The axial fast current has an average current of ~ MA, which generates the strong azimuthal magnetic field of the order of GGauss. Upon exiting the channel, the magnetic field expands into vacuum and the electron current is dissipated. This field has the form of a dipole in 2D and a toroidal vortex in 3D. The magnetic field displaces the electron component of plasma with regard to the ion component and a strong quasi-static electric field is generated at rear side of plasma target which accelerate and collimate the ion beam to achieve higher energies. The effectiveness of this mechanism depends on the efficient transfer of laser pulse energy into the energy of fast electrons that are accelerated along the laser propagation direction. Thus in MVA mechanism for each laser target configuration there exists an optimum target thickness that maximizes the ion energy.

In this report, we demonstrate that an ultraintense-short CP laser pulse generates a ‘relativistic electron beam of helical shape’ and ‘collimated proton beam’ from NCD hydrogen plasma via MVA mechanism. Simulation results show quasi-monoenergetic feature in energy spectra of electron and proton beam due to interaction of CP laser pulse with near critical density plasmas (~1.0 n_c_). The extensive 3D PIC simulation results show high laser-to-proton conversion efficiency with peak proton energy 350 MeV. We extended further the MVA ion acceleration in non-uniform plasma and demonstrated the role of density scale length at rear side of plasma-vacuum interface on ion acceleration. The results highlighted the fact that high-energy ion acceleration can be realised in experiment by using the near-critical density target with optimum plasma thickness and by controlling the laser beam parameter.

## Results

**Figure 1:**
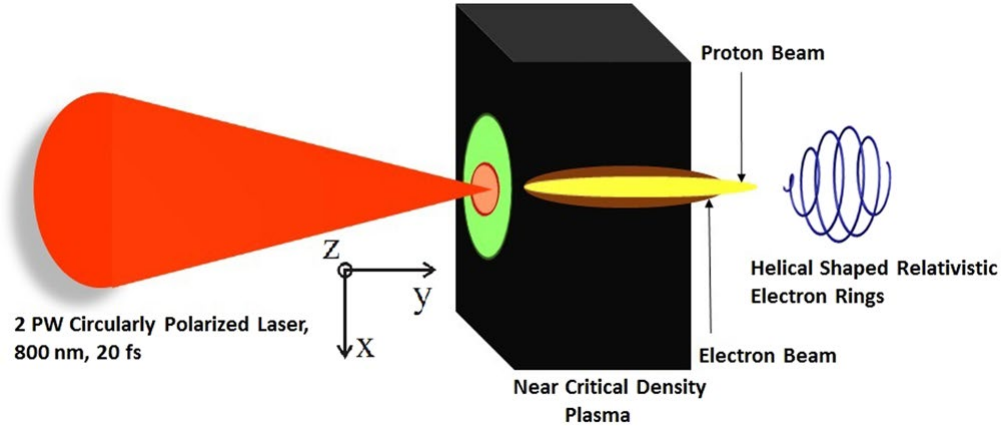
Sketch of the setup for the interaction of a circularly polarized PW laser pulse (2PW, 800 nm, 20 fs) with a pre-ionized near critical density plasma target. Shown by black slab is a target with uniform plasma density (*~1*.*0 n*_*c*_). Blue spiral curve shows the generation of helical relativistic electron beam from MVA mechanism.

Starting with the interaction of a CP laser pulse propagating in the NCD hydrogen plasma along the Y-direction. The schematic of CP laser interaction with the target is shown in Fig. 1. The plasma target considered in this simulation study is uniform and non-uniform both. The electric field of laser can be expressed as *E*_*L*_(*r*, *y*, *t*) = (1/2)*E*_0_(*r*, *y*, *t*)(*e*_*x*_ + *ie*_*z*_)*exp*(−*iωt* + *iky*) + *c*.*c*. The laser pulse propagates with the group velocity, *v*_*gr*_ = *c*^2^*k*/*ω* where *k* = 2*π*/*λ* is the wave vector given by the dispersion relation ${c}^{{\rm{2}}}{k}^{{\rm{2}}}={\omega }^{2}-{\omega }_{p}^{{\rm{2}}}$. The spatio-temporal intensity profile of laser is considered as Gaussian distribution and can be written as $I={I}_{0}\exp (-{r}^{{\rm{2}}}/{r}_{{\rm{0}}}^{{\rm{2}}})\exp (-{t}^{2}/{\tau }_{0}^{2})$, where *r*_0_ is the transverse radius of laser beam and *τ*_0_ is laser pulse duration. For optimum acceleration in case of MVA^23^, the laser spot size should match the size of the self-focusing channel in order to avoid filamentation. The laser beam radius in an underdense plasma can be written as $r={r}_{0}{[{\rm{1}}+({x}^{2}/{x}_{R}^{2})({\rm{1}}-(P/{P}_{C})]}^{{\rm{1}}/{\rm{2}}}$ where ${P}_{c}={\rm{17.5}}({n}_{c}/{n}_{e})GW$ is the critical power and ${x}_{R}$ is the Rayleigh range.

The effectiveness of MVA mechanism requires the efficient transfer of laser energy to the fast electrons in the plasma, which are accelerated in the plasma channel along the laser propagation direction. The optimum plasma length^23^ ${L}_{ch}={a}_{L}c\tau ({n}_{c}/{n}_{e})K$ can be estimated from the assumption that all laser energy is transferred to the electrons in the plasma channel, where *K* is the geometry constant (*K* is 0.1 in 2D case and 0.074 in 3D case), ${a}_{L}=e{E}_{L}/{m}_{e}{\omega }_{{\rm{0}}}c$ is the normalised electric field amplitude of laser, *e & m*_*e*_ are fundamental charge and electron mass, *E*_*L*_ is the laser electric field, *ω*_0_ is the laser frequency and c is the speed of light in vacuum.

**Figure 2:**
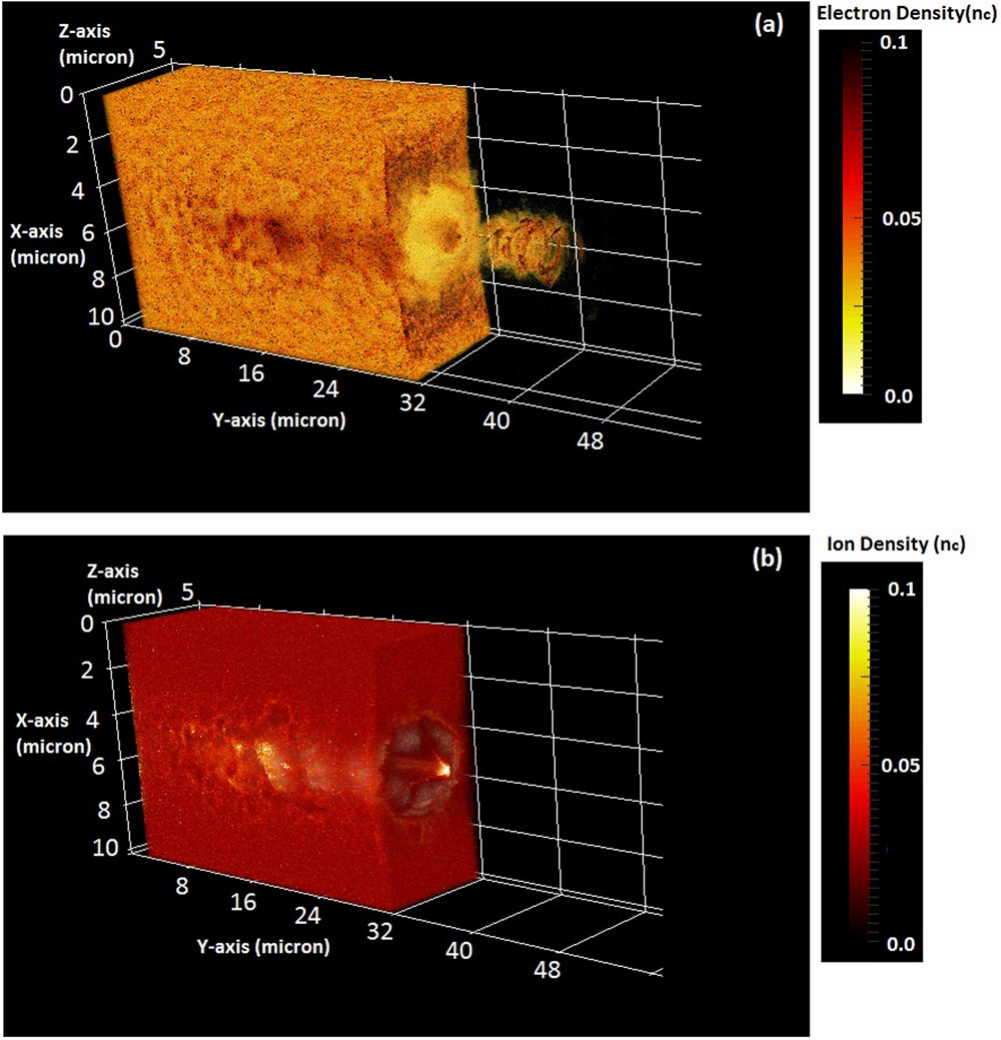
Evolution of electron (**a**) and ion (**b**) charge density at time instant 200 fs. The laser amplitude, pulse duration, and beam radius are a_0_ = 81, τ_0_ = 20 fs and r_0_ = 1.5 µm respectively. The density of hydrogen plasma target is n_e_ = n_c_ and the thickness of plasma target is L_target_ = L_ch_ = 30 µm. Color bars show the variation in electron and ion charge density.

We begin by performing the simulation for the idealized case of plasma target – that is, with sharp plasma-vacuum interface – and CP laser. Figure 1 shows three dimensional (3D) particle-in-cell (PIC) simulation set-up where a 2 PW CP laser pulse with a pulse length 20 fs (FWHM) and focal spot diameter of 3 µm (FWHM) is focused on a plasma slab of uniform plasma density and of thickness 30 µm. Detailed simulation parameters can be found in the Methods. We show in Fig. 2, the density distribution of the electron (a) and ion (b) charge density at time instant when the depleted laser pulse transmits from the rear side of plasma target. By this time the laser pulse has been passed through the NCD plasma target and, has formed a channel from which the electrons and ions are expelled preferentially in the transverse direction to the laser propagation direction. Since the normalized laser field (a_0_ = 81) of petawatt laser is much larger than the value of (square root of ration of ion mass to electron mass) (*m*_*i*_/*m*_*e*_)^1/2^, which means that the physical processes due to nonlinear ion dynamics come into role where we may not neglect the ion plasma dynamics with regard of electron plasma motion in defining the nonlinear dielectric function of plasma.

The most interesting point here is the observation of relativistic electron beam of helical shape in vacuum at rear side of plasma target. Figure 2a shows the helical electron beam at time instant 200 fs when depleted laser pulse exits from the rear side of plasma. To understand the formation of helical shape electron beam, we followed the interaction of focused laser pulse in underdense plasma channel, which generates the axial and azimuthal magnetic field^33^. Axial (*B*_*y*_) and azimuthal magnetic field (*B*_*z*_) can be written as respectively,

1 $$\frac{\partial {B}_{y}}{\partial r}=\frac{-\alpha }{{\rm{1}}+{\alpha }^{2}}{n}_{e}\frac{{\rm{1}}}{\gamma }\frac{\partial }{\partial r}(\frac{\langle \delta {n}_{e}\rangle }{{n}_{{\rm{e}}}}\frac{{\gamma }^{{\rm{2}}}-{\rm{1}}}{\gamma })$$

2 $$\frac{{\rm{1}}}{r}\frac{\partial }{\partial r}(r{B}_{Z})=-{n}_{{\rm{e}}}\frac{\delta {n}_{e}}{{n}_{{\rm{e}}}}\frac{\gamma -{\rm{1}}}{\gamma }$$

where *γ* is the relativistic factor, *δn*_*e*_ is the modulation in electron density due to the laser field and can be determined as $\delta {n}_{e}/{n}_{e}=1+({e}^{2}/4{m}_{e}^{2}{\omega }_{p}^{2}{\omega }^{2}\gamma ){\nabla }^{2}\langle {\boldsymbol{E}}\cdot {E}^{\ast }\rangle$, *α* = 0(*LP*), ±1(*CP*) and $r=\sqrt{{x}^{2}+{z}^{2}}$ is the radius of channel in cylindrical coordinate system. The expression for axial magnetic field (*B*_*y*_) (as given by Eq. 1) shows that it can be generated only by CP laser however in case of LP laser the axial magnetic field is zero. The azimuthal magnetic field (given by Eq. 2) can be generated from the longitudinal current driven by the ponderomotive force of CP and LP laser both.

Thus it is evident that due to radial spatial field dependence of focused circularly polarized laser there will be an axial magnetic field ${\overrightarrow{B}}_{y}$, which in turn leads the generation of $({\overrightarrow{v}}_{x,z}\times {\overrightarrow{B}}_{y})$ force in transverse direction of the same order as the transverse component of the ponderomotive force. Consequently a symmetric, tightly focused laser will tend to eject ring of electrons modulated in helical shape (as shown by Fig. 2a) in laser propagation direction.

**Figure 3:**
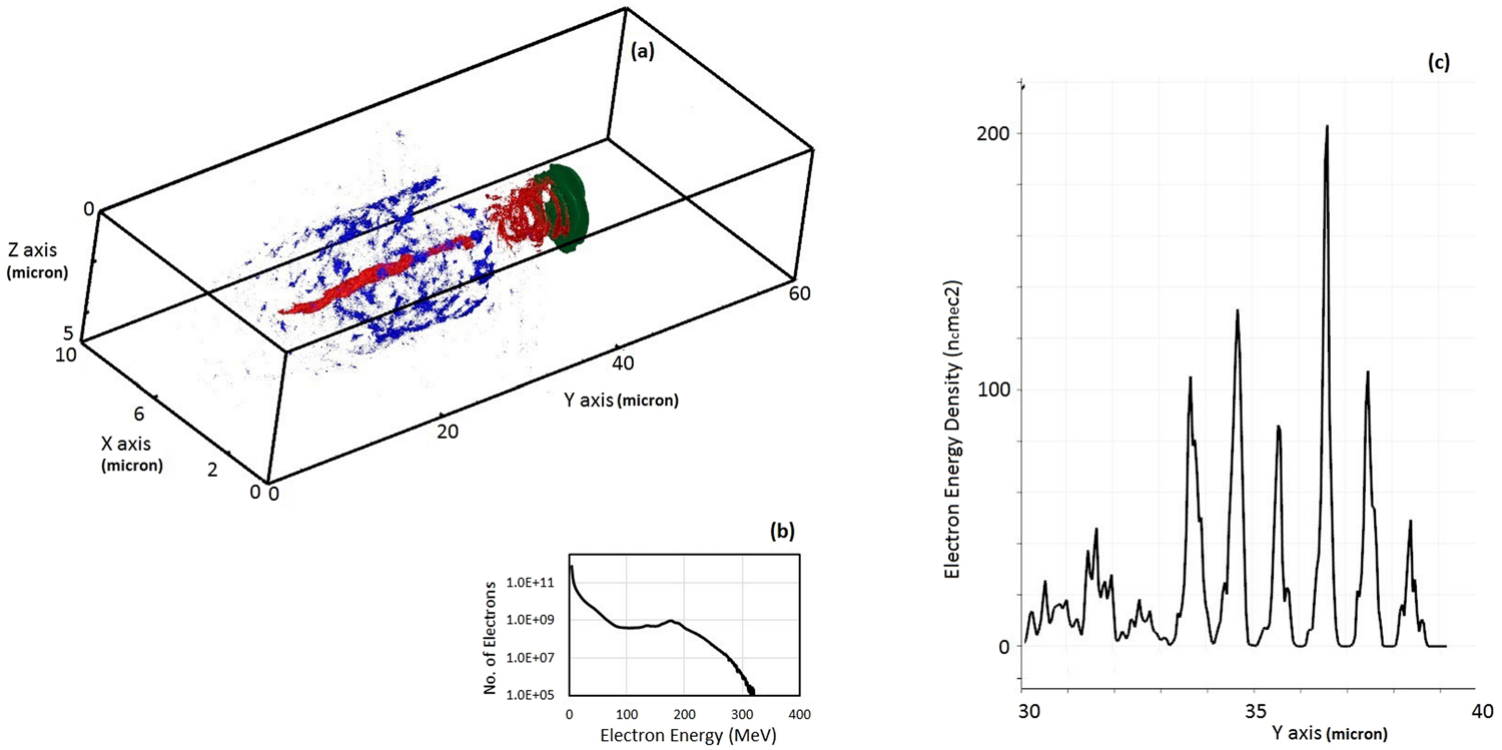
(**a**) Contour plot of forward current (red) and return current (blue) in plasma channel which is followed by depleted laser pulse (green); forward electron current of helical beam can be seen which is trailing just behind the pulse, (**b**) quasi-monoenergetic energy spectrum of electrons, and (**c**) Energy density plot of helical electron beam: distribution of relativistic electrons (corresponding to length of helical electron beam) along the laser propagation direction (Y-axis) length. The energy density of electrons are normalised with n_c_m_e_c^2^. The simulation results shown are for laser – plasma parameters as shown in Fig. 2.

We show here in Fig. 3a, the helical shaped electron beam by depicting the return current (blue) and forward current (red) which is trailing behind the laser pulse (green). Helical electron beam shown in red shows the ring structures with negligible axial current. The contour plot shown in Fig. 3a represents the peak value of forward and return current as 0.3 en_c_c and peak value of electric field ~10^14^ V/m. Figure 3b shows the electron energy distribution at time instant, after depleted laser pulse exits from the rear side of plasma and maximum acceleration is achieved. The energy spectrum of electron beam shows the quasi-monoenergetic feature due to CP laser, which is different from typical thermal energy distribution. More interestingly mono-energetic feature is traced on energy distribution of proton beam (depicted later for proton beam characteristics). The energy density distribution in Fig. 3b corresponds to the electrons of helical shaped electron beam along the propagation direction of laser, which characterised this electron beam. Helical shaped high-energy electron beam^34,35^ with quasi-monoenergetic nature may be used for plasma based X-ray source and collimation of positron beam source. Simulation Results on generation of helical shaped high-energy electron beam from MVA mechanism has shown resemblance with the previous experiment and simulation study^35^ where relativistic energy electron rings were observed from laser wakefield acceleration experiments in the blowout regime. The ring produced in both the experiment and simulation were accompanied by substantial axial current making it impractical; to utilise these rings for applications the ratio of the ring to axial current must be increased. In this report, we find that magnetic vortex field at the rear side of target prevents the flow of axial electron current resulting in hollow electron beam of helical shaped rings. The results shown in Figs 2(a) and 3(a) for electron charge density and current distribution illustrates this feature where axial electrons are stopped due to magnetic field and helical shaped hollow electron rings can be seen propagating in vacuum.

As shown previously (Figs 2a–3a) that high-energy electrons are localised along the centre axis of channel and a fraction of them are accelerated in forward direction, which trail behind the laser front, forming an axial fast current. To maintain plasma quasi-neutrality, a cold electron return current is formed to balance the fast current. Thus, background electrons flow outside the channel forming a return current.

**Figure 4:**
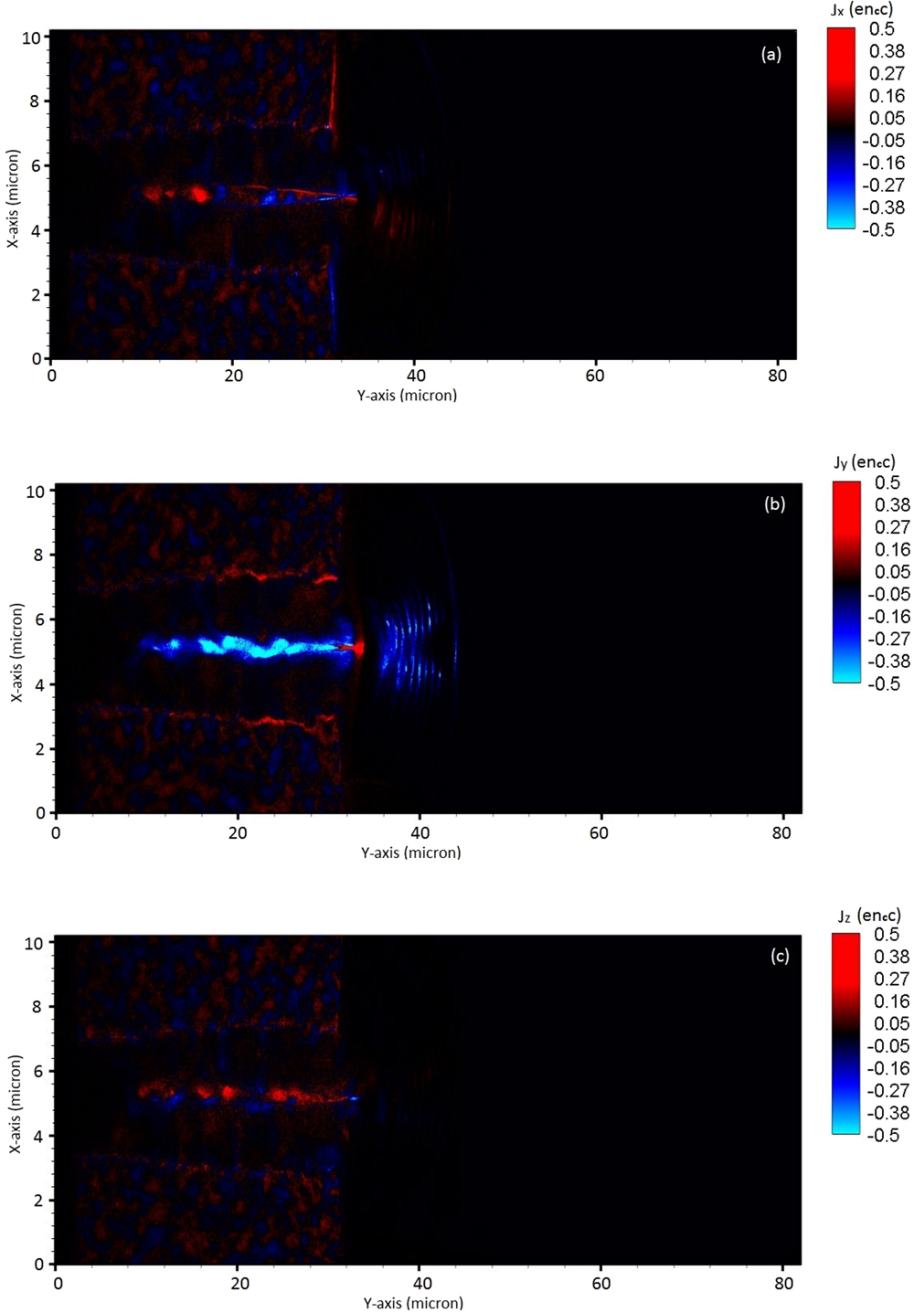
The contour plot (**a–c**) of current density (J_x_, J_y_ and J_z_) (normalised with e n_c_ c) along the channel in laser propagation direction at time instant 200 fs. The simulation result shown are for laser – plasma parameters as shown in Fig. 2. X-axis and Y-axis are in micron and colour bar shows the variation in current density.

We show in Fig. 4, component of current density distribution along x, y, and z direction. It can be seen from Fig. 4b that forward current density (blue) is dominating near the axis and the return current (red) dominates away from the axis along the channel wall. Its evident from Fig. 4a–c two oppositely signed currents repel each other and subsequently forming an axial fast current and cold return current. The on axis fast current has an average current density ~ *n*_*c*_*ec* which leads to the generation of large azimuthal magnetic field. In case of homogenous plasma at plasma – vacuum interface, the abrupt decrease in magnetic field induces a strong electric field, which is responsible to accelerate the ion beam to high energies.

**Figure 5:**
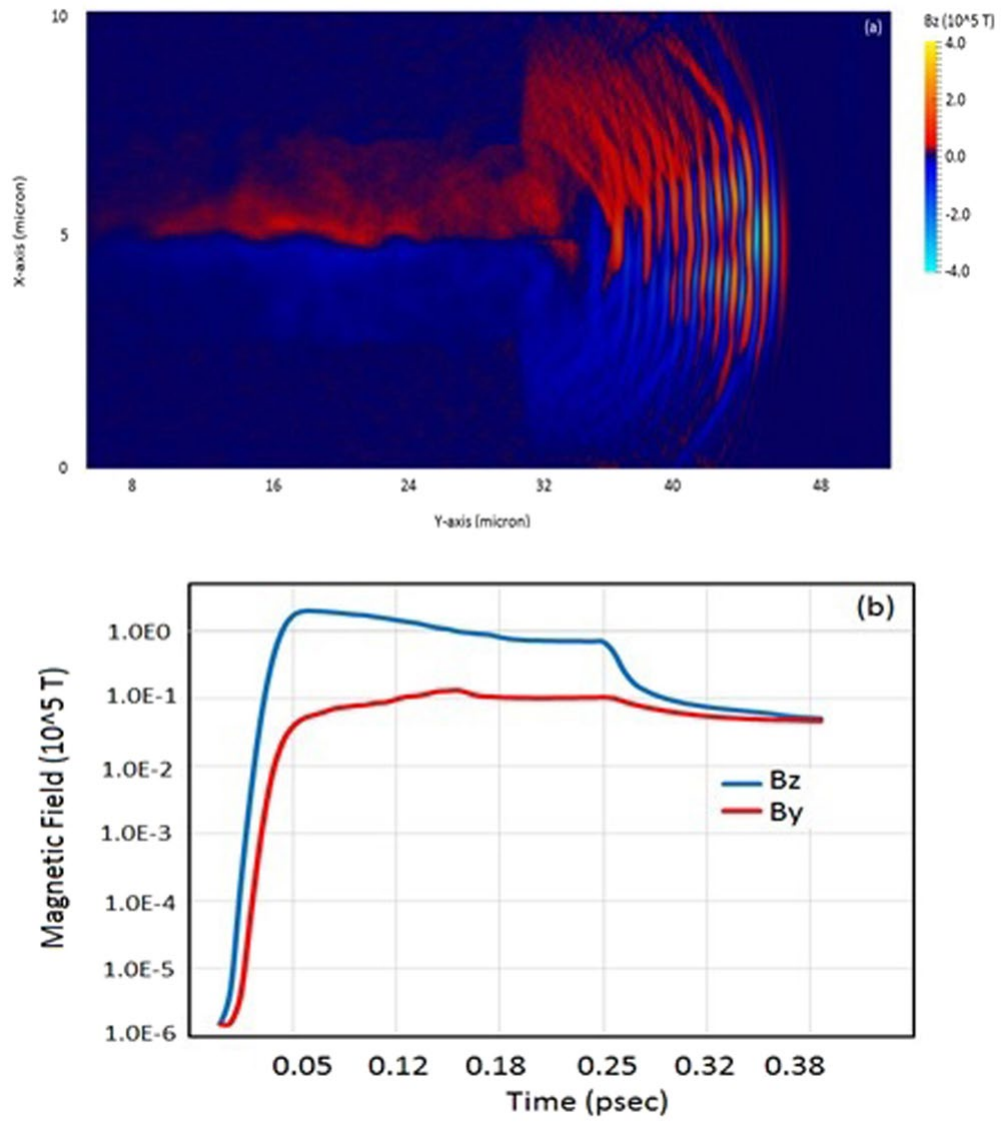
Spatial (**a**) and temporal (**b**) evolution of self – generated plasma magnetic field from the simulation after the laser exits the plasma at 200 fs. The simulation results shown are for laser – plasma parameters as shown in Fig. 2.

We estimated the axial and azimuthal magnetic field using the analytical expression (from Eqs 1 and 2) for the laser-plasma parameters as detailed in ‘Method’. It is found from the analytical estimation that axial magnetic field is peaked near the laser axis (i.e. *r* = 0 which corresponds to the centre of channel) and dominates the inner zone which is peaked about $|{B}_{y}|$ = 10^4^ *T*. Azimuthal magnetic field observed from analytical estimation is estimated about ~10^5^ *T* which is dominating the outer zone. The simulation results show that generated azimuthal magnetic field and axial magnetic field for the laser-plasma parameters adopted here is of the order of 10^5^ *T* and 10^4^ *T* respectively (as shown by Fig. 5b). The simulation results for *B*_*z*_ (~10^4^ *T*) of Bulanov^24^ is consistent with the results shown here and an order of magnitude lower due to different laser-plasma parameters.

Figure 5a, b shows spatial and temporal evolution of azimuthal and axial magnetic field at time instant when laser pulse exits the rear side of target. Figure 5b shows the peak value of *B*_*y*_ and *B*_*z*_ which is estimated from simulation results at each time step for simulation time 0 fs to 400 fs; 400 fs correspond to the time instant after the maximum acceleration reached to saturation.

**Figure 6:**
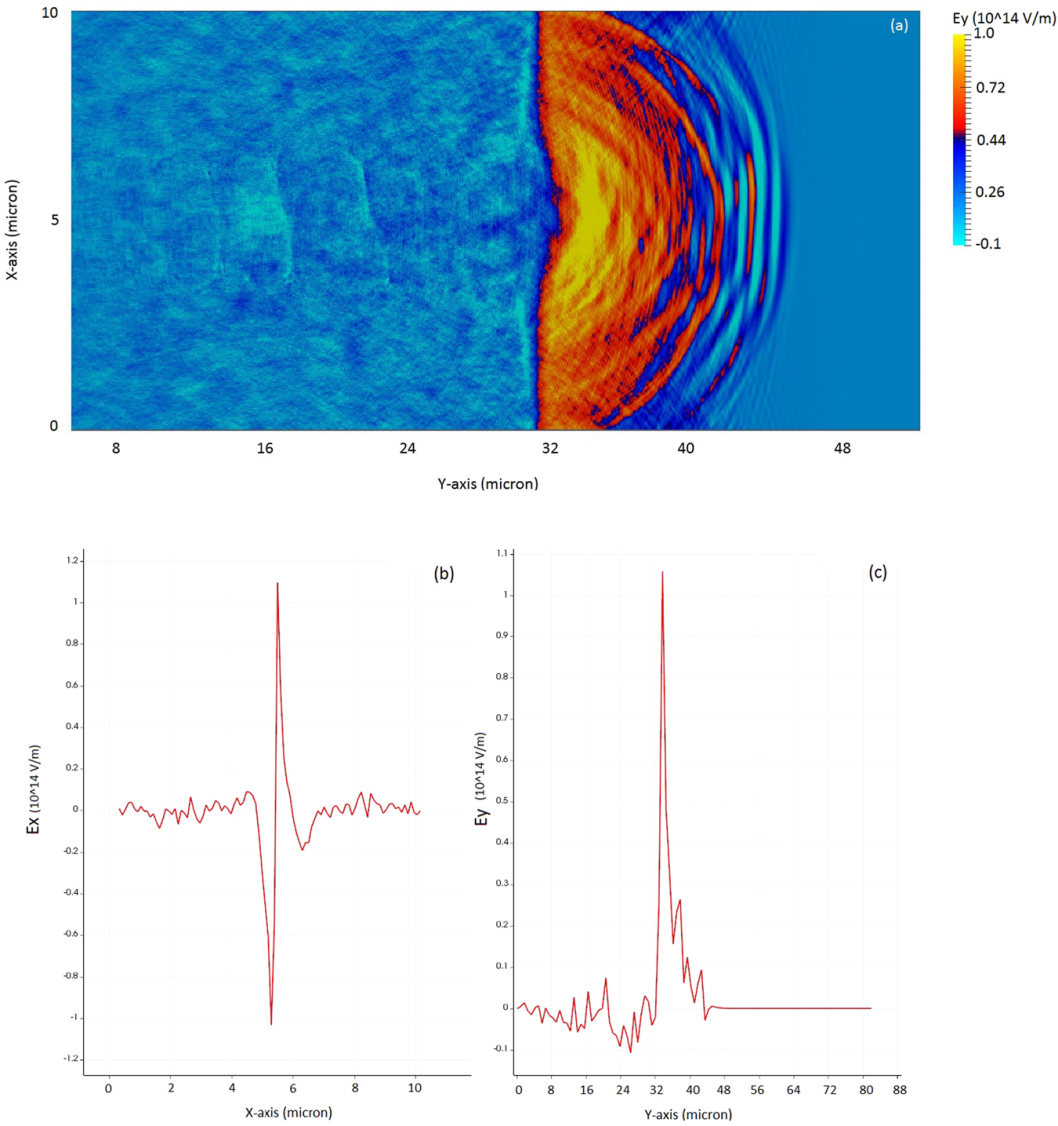
The spatial evolution of self – generated plasma electric field (**a**) and evolution of transverse focusing field normal to the laser propagation direction (**b**) and along the laser propagation direction (**c**); after the laser exits the plasma at 200 fs.

As shown in Fig. 5(a), as the laser pulse exits the plasma, the azimuthal magnetic field expands longitudinally and transversally as well. The transverse and longitudinal expansion of azimuthal magnetic field leads to the decrease of electrons drift speed, which are coming out from the plasma channel. Thus the slowdown of electron speed increases the density locally and hence the current distribution at rear side of target. In our study the transverse and longitudinal expansion of azimuthal magnetic field (shown by Fig. 5a) is playing a key role at the rear end of plasma target; to determine the accelerating field (shown by Fig. 6) and hence acceleration of ions/deceleration of electrons. We show in Fig. 6, the spatial distribution of induced electrostatic field, which is peaked at plasma – vacuum interface as, can be seen from Fig. 6c, the spatial variation of longitudinal accelerating field along the laser propagation direction. Figure 6b shows the variation of transverse focusing field, which is responsible for ion beam focusing towards the propagation axis.

We illustrated the MVA mechanism (as depicted in Figs 2–6) which relies on self-generated quasi-static magnetic field (Fig. 5) and electric field (Fig. 6) at rear side of plasma for efficient forward ion acceleration and collimation. Here in CP laser pulses driven MVA, we have shown that magnetic pressure expels electrons from the magnetic region into the plasma channel and builds up an electrostatics field, which accelerates the ions in laser propagation direction at plasma-vacuum interface.

**Figure 7:**
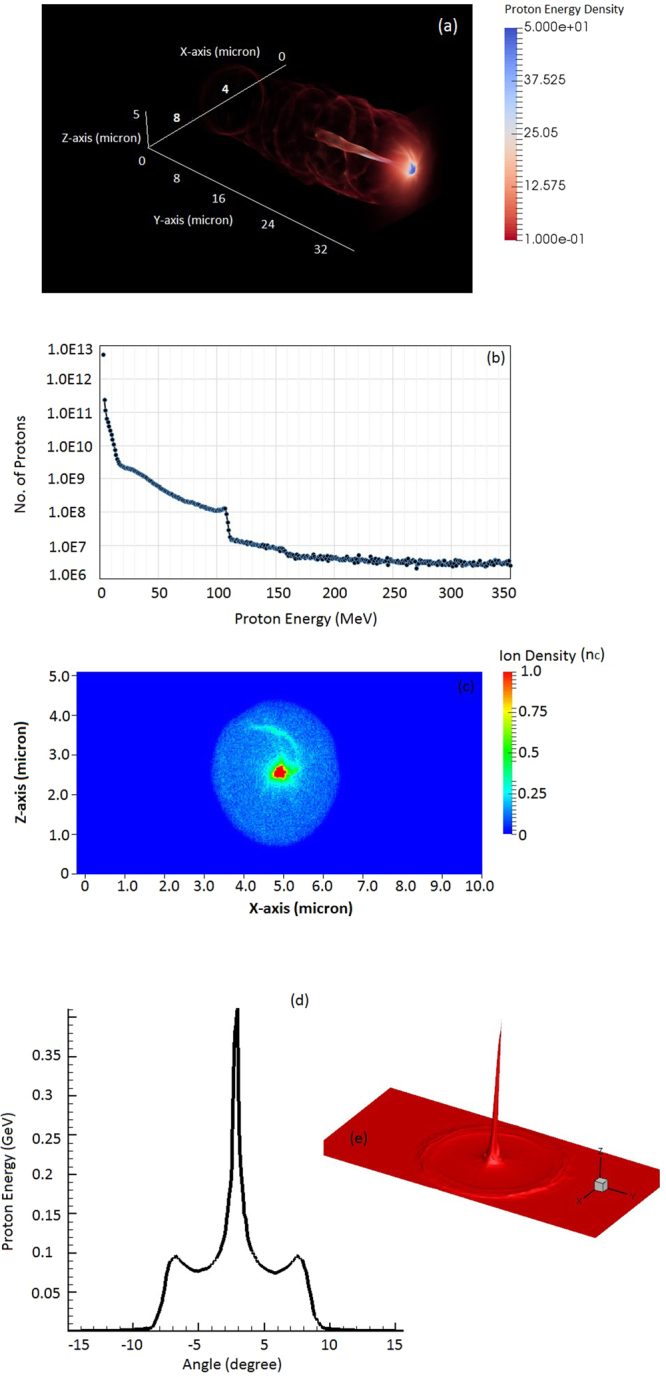
(**a**) The distribution of proton energy density, normalised with n_c_m_e_c^2^. Energy (**b**) and density (**c**) distribution of protons at time instant 200 fs. The simulation results shown are for laser – plasma parameters as shown in Fig. 2. The angular distribution of protons is shown by (**d**, **e**).

We depicted the proton energy distribution in Fig. 7 at time instant 200 fsec when the laser pulse exits the plasma channel. Further the proton beam is characterised (as shown in Fig. 7b,c) by showing the energy and spatial density distribution at time instant of maximum acceleration. Figure 7b shows the energy distribution of protons with maximum proton energy about 350 MeV. Figure 7c shows the spatial density distribution of focused proton beam where high-energy protons are concentrated in very small area; which can be attributed to transverse focusing field as shown in Fig. 6b. Figure 7(d,e) shows the angular distribution of proton beam, which clearly demonstrates that high-energy protons are collimated at smaller divergence angle.

A spectral peak at 100 MeV has been shown in proton energy spectrum (as in Fig. 7b) which is mapped from quasi-monoenergetic spectrum of electrons (as shown by Fig. 3b). The spectral peak in energy spectrum of electrons/protons is observed due to the different transit time of fast (slow) electrons/protons with respect to the transient longitudinal electric field.

Since, typical gas-jet targets have linear or parabolic density scale-lengths; too long for the production of quasi-monoenergetic ion beams^36,37^. Recent experiment^26^ demonstrated the multi-MeV proton acceleration from the ‘gas foil’ target, available at near critical density at near infrared wavelengths but with the density ramp on both sides. Therefore it’s necessary to investigate the role of density ramp in ion acceleration mechanism and to approximate the dependence of peak energy of ions on plasma density gradient.

**Figure 8:**
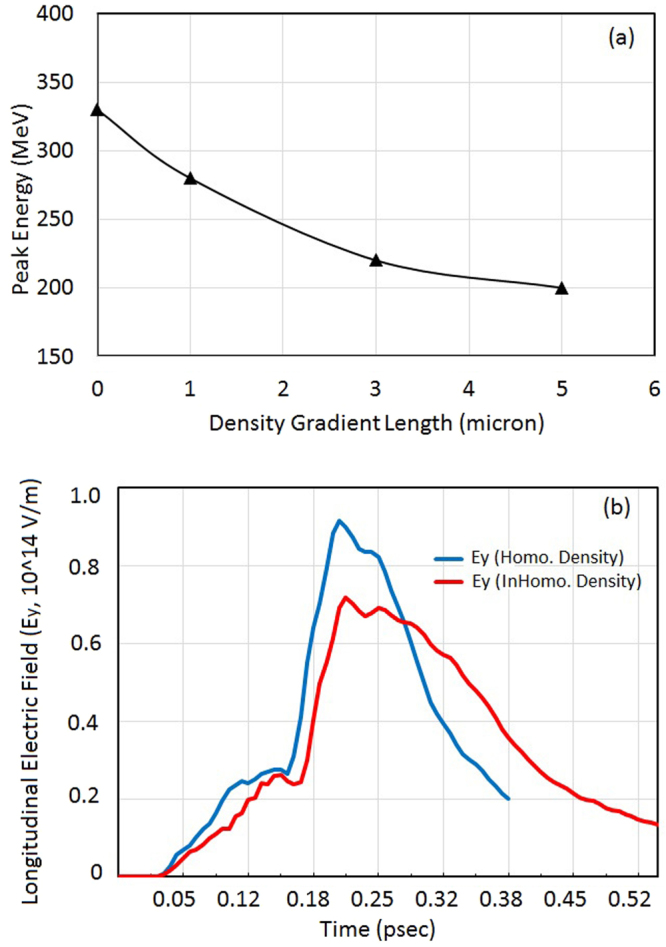
Dependence of peak proton energy on density gradient (**a**) and evolution of longitudinal electric field (**b**) with time. The longitudinal field evolution explains the dependence of peak proton energy on density gradient. The simulation results are shown at the stage of maximum ion acceleration (after 200 fs). The simulation results shown are for laser – plasma parameters as shown in Fig. 2.

The dependence of peak proton energy on density scale length is shown in Fig. 8a; which is obtained in simulation by considering plasma with density gradient at front and back side of target. The length of flat part of the plasma target is considered as 30 µm while the ramp length is varied 0 (homogenous plasma), 1, 3 and 5 µm to study the role of plasma density gradient at front and rear side of target. The induced electrostatic field responsible for ion acceleration depends on charge separation between ions and electrons. The electrons moving realistically in the forward direction are driven by the magnetic vortex field. The lateral expansion velocity of the magnetic vortex can be determined from density scale length of plasma and the density scale length can be expressed as $L=|{n}_{e}/(d{n}_{e}/dx)|$. A large density gradient result in the faster lateral expansion of the vortex and the vortex has no time to form density spike and subsequently magnetic vortex acceleration has less influence on ion acceleration. Thus, this study also suggests that to get more ions that are energetic in the forward direction, one has to enhance the longitudinal electric field, for example by using targets with much steeper density gradients. Figure 8b shows the comparative dependence of induced longitudinal field with time for homogenous (with ideal condition of plasma – vacuum interface) and inhomogeneous plasma target (with density gradient at front and rear side of plasma target). As per theoretical estimation, simulation results also show the decrease in longitudinal field in case of plasma density gradient, which may affect the proton acceleration to obtain higher peak energy.

## Conclusion

In conclusion, we demonstrated the generation of quasi-monoenergetic helical shaped relativistic electron ring in MVA mechanism employing the CP laser pulse interaction with the near critical density plasma. These relativistic electron rings have shown the possibility of a relatively cheap and compact accelerator/collimator for ultrahigh energy protons source as shown via numerical results; which were originally proposed for the collective acceleration of ions in conventional (RF) accelerators^33^.

We have shown further MVA driven proton acceleration where energetic protons with peak energy 350 MeV, charge ~10 nC and conversion efficiency more than 6% (which implies 2.4 J proton beam out of the 40 J incident laser energy), as estimated from the simulation outcome shown by Fig. 7. In our previous research^23^, we demonstrated the proton acceleration via MVA mechanism with tightly focused LP laser pulse (2PW-20fs) interaction with near critical density plasma (~3.0 n_c_). Simulation results in this regime shows comparatively lower laser-to-proton conversion efficiency ~1% (for protons with energy > 4 MeV) but higher peak proton energy ~GeV.

More interestingly, the quasi-monoenergetic feature of electron energy spectrum can be observed in proton energy spectrum as well. The role of plasma density gradient has also been examined in order to model the gas target for ion acceleration experiment. Simulation results supported by theoretical model in this work proposed the near critical density target with sharp plasma-vacuum interface or steeper density gradient for efficient acceleration to be realised.

Thus, simulation study suggests the laser-plasma parameters for experimental illustration in order to achieve high proton energy and higher number of protons by employing near critical density target interaction with the CP laser pulses. The proposed density window is preferred in order to get experimental access of near critical density gas targets^26^, which may allow generating the high flux of ions by employment of high rep. rate solid-state PW lasers^38^. In recent time few Petawatt laser systems^38^ are already in operation and advanced laser facilities such as, Extreme Light Infrastructure (eg. ELI-ALPS)^39^, will be able to provide laser pulses with several Petawatts. The availability of PW laser system may allow us to achieve focused intensities >10^22^ W/cm^2^. However perfect focus-ability of multi-Petawatt laser pulses may be difficult to observe, and halo problem^40^ can be a key issues for the desired outcome in the experiments.

## Methods

### Particle-in-Cell Simulations

To illustrate efficient ion acceleration and underlying microphysics involved in it; the simulations were performed using the relativistic 3D PIC code, PIConGPU^41^. Each simulation was defined with Cartesian spatial dimensions of 10 × 100 × 5 µm^3^. A circularly polarized laser pulse (800 nm wavelength laser) enters the simulation box from the left boundary propagating along the y-direction. A Gaussian temporal profile of 20 fs (FWHM), focused to a Gaussian spatial intensity profile of 3 µm (FWHM). The normalized laser electric field amplitude is ${a}_{L}=e{E}_{L}/{m}_{e}{\omega }_{{\rm{0}}}c$ = 81. Simulations were performed for a total duration of 400 fs to ensure full propagation of the laser pulse through the simulation space.

The target in each simulation is representing hydrogen plasma of density *n*_*i*_ = *n*_*e*_ = 1.0*n*_*c*_ (critical density *n*_*c*_ = 1.74 × 10^21^ cm^−3^). We used 40 cells per wavelength in the x-direction, 400 cells per wavelength in the y-direction (direction of laser propagation) and 40 cells per wavelength in z-direction in the simulation. The time step in these computationally intensive 3D simulations is 66.7 asec. Two simulation particles per cell for each species are considered in the simulation.

Through the set of simulation runs, we investigate the relevance of circular polarisation, effect of density gradient (by varying the density scale length from 0 to 5 micron) and influence of self-generated electric and magnetic field on ion beam characteristics.

## Electronic supplementary material

Supplementary material
