# Supplementary material for: High Energy electron and proton acceleration by circularly polarized laser pulse from near critical density hydrogen gas target
Source: Sci Rep. 2018 Feb 1;8:2191. doi: 10.1038/s41598-018-20506-x (PMC5794773; doi:10.1038/s41598-018-20506-x)
Supplement: Supplementary file 1 — Supplementary material [file 41598_2018_20506_MOESM1_ESM.pdf]

Supplementary material

on

High Energy electron and proton acceleration by circularly polarized laser pulse from near critical density hydrogen gas target

ASHUTOSH SHARMA

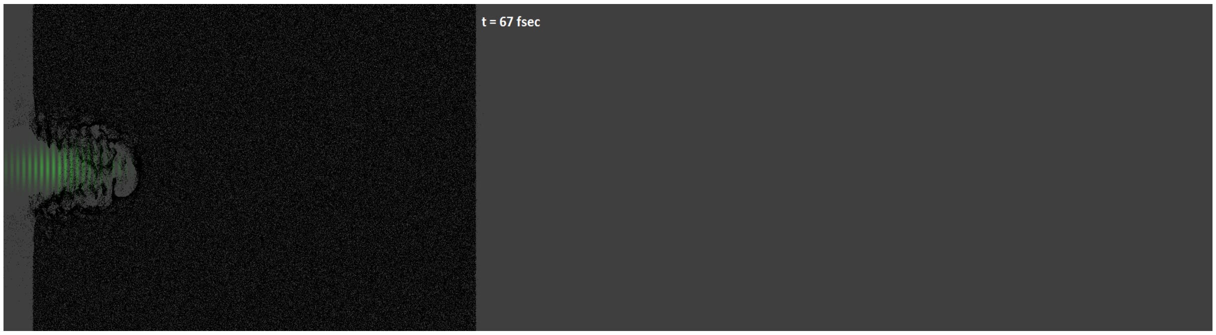

(a)

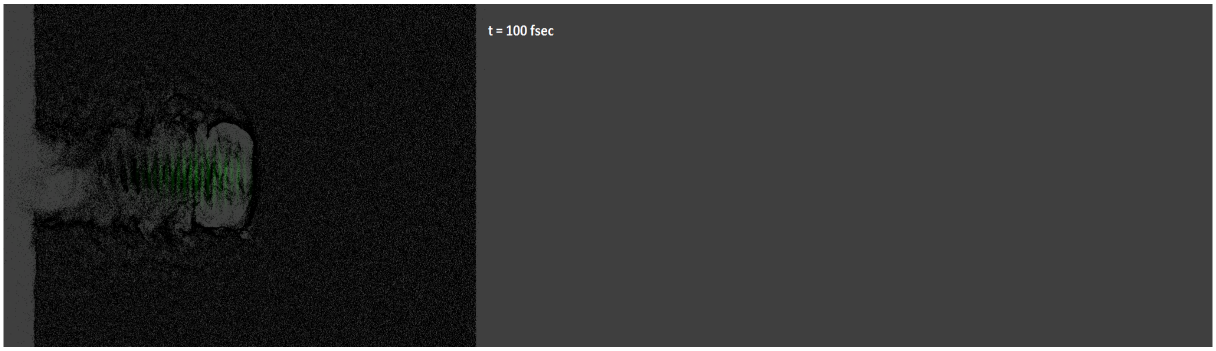

(b)

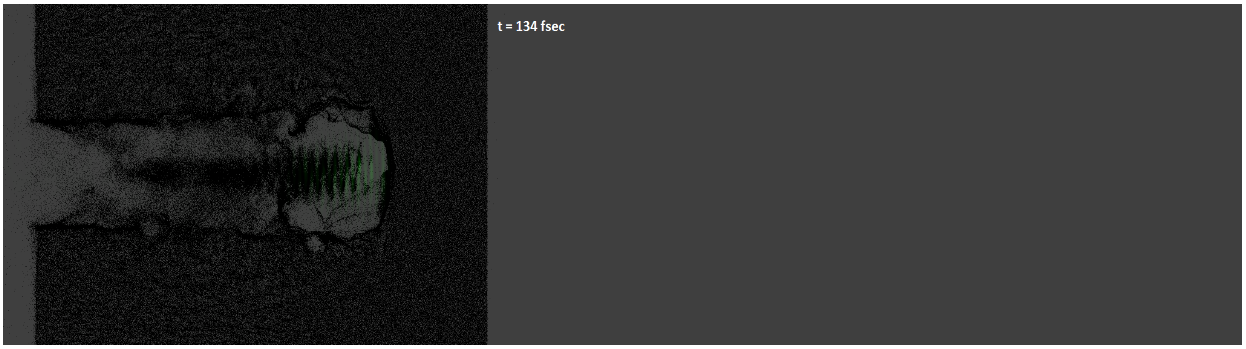

(c)

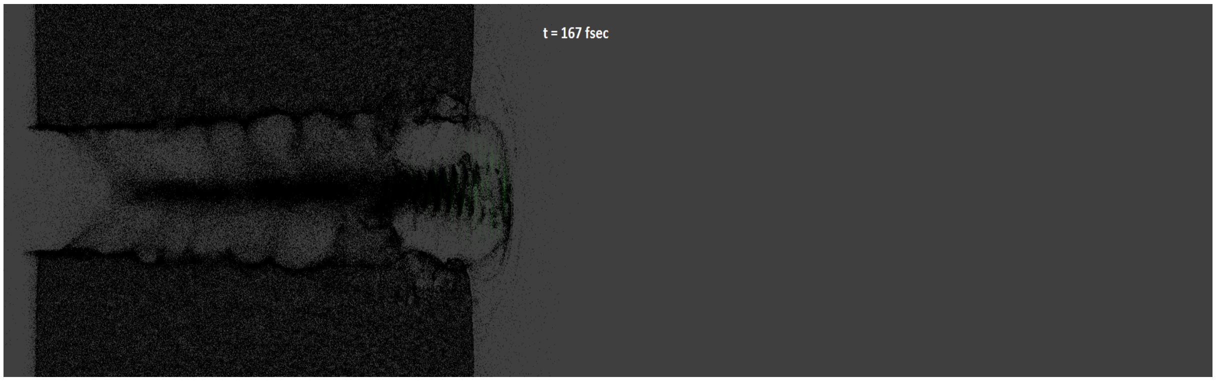

(d)

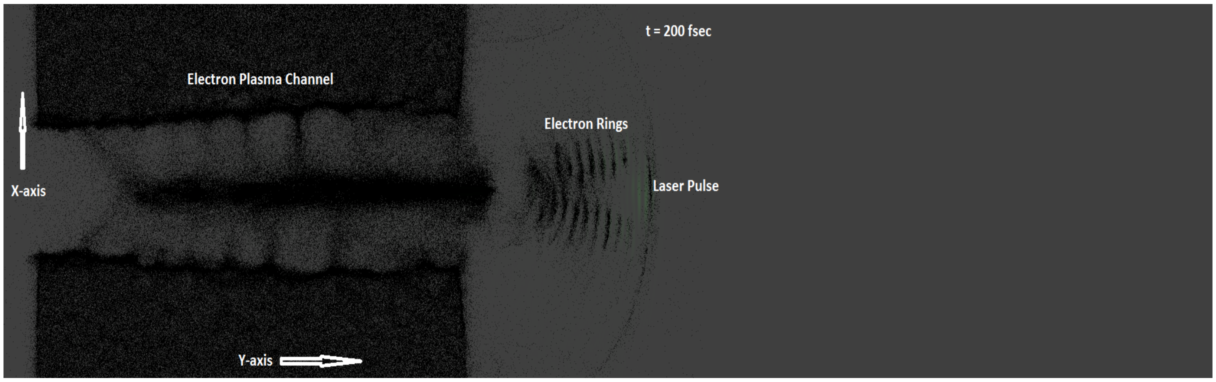

(e)

Figure S1: Evolution of electron density distribution in XY plane at different time instants, (a) 67fs, (b) 100fs, (c) 134fs, (d) 167fs and (e) 200fs. Black colour corresponds to the electron density distribution and green colour is shown for laser pulse.

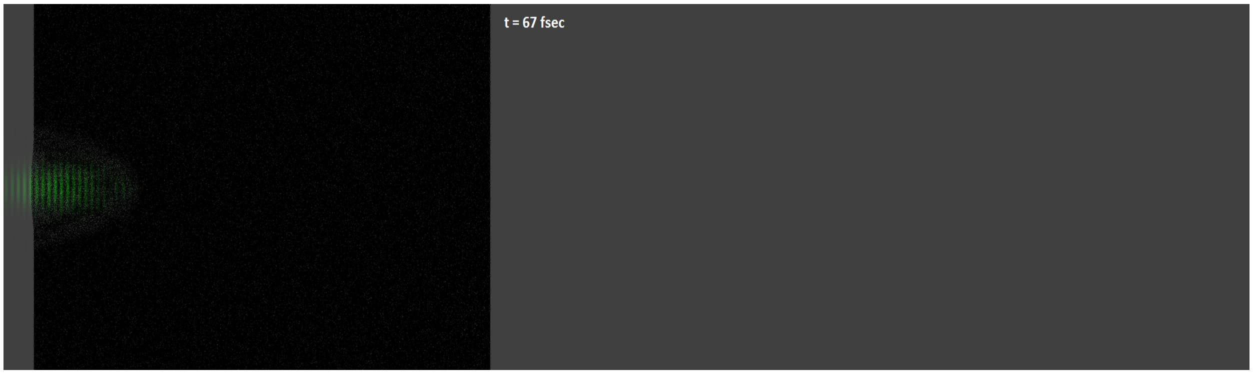

(a)

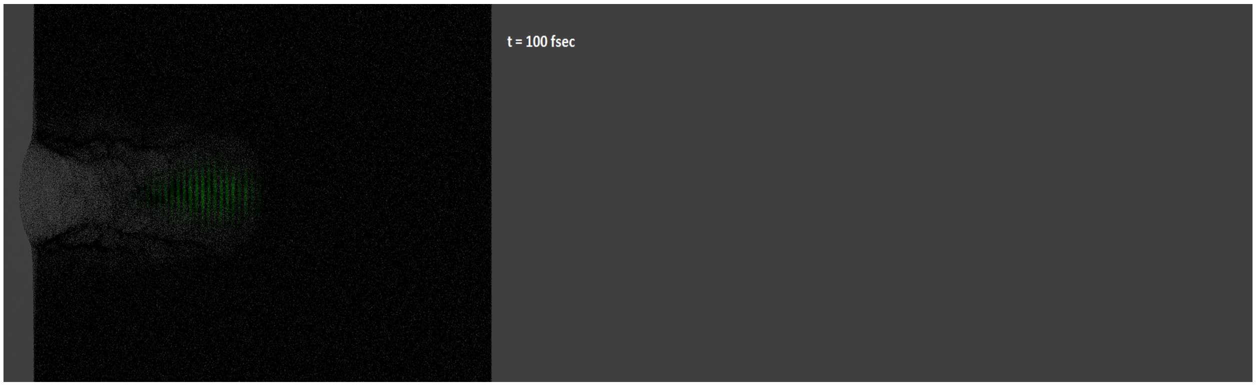

(b)

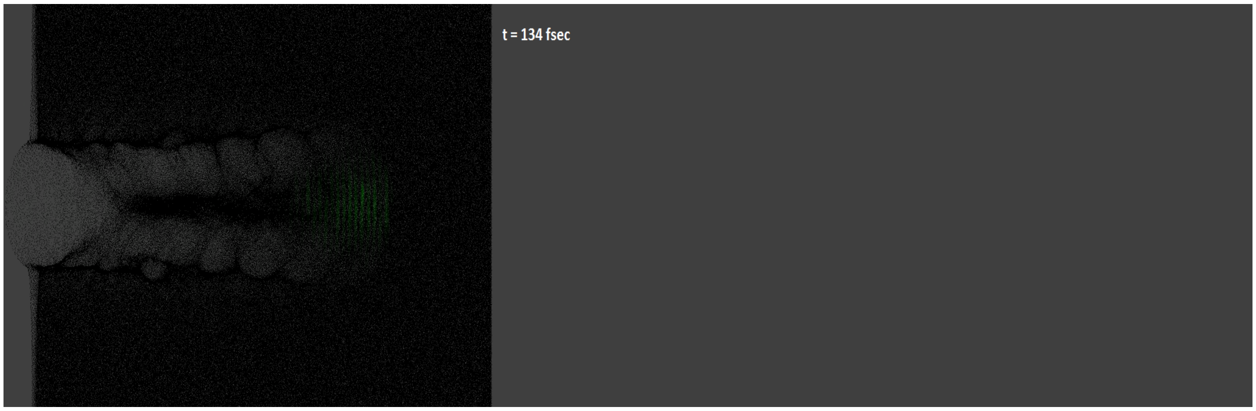

(c)

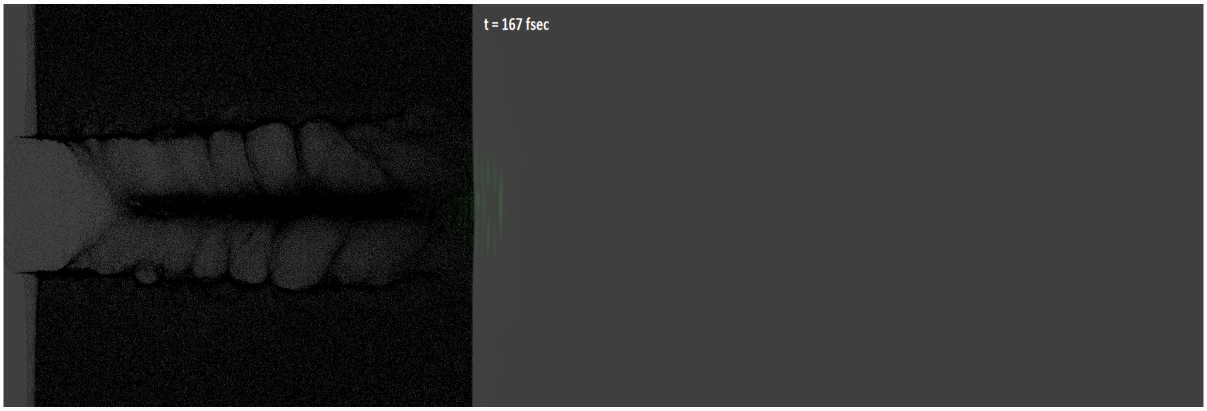

(d)

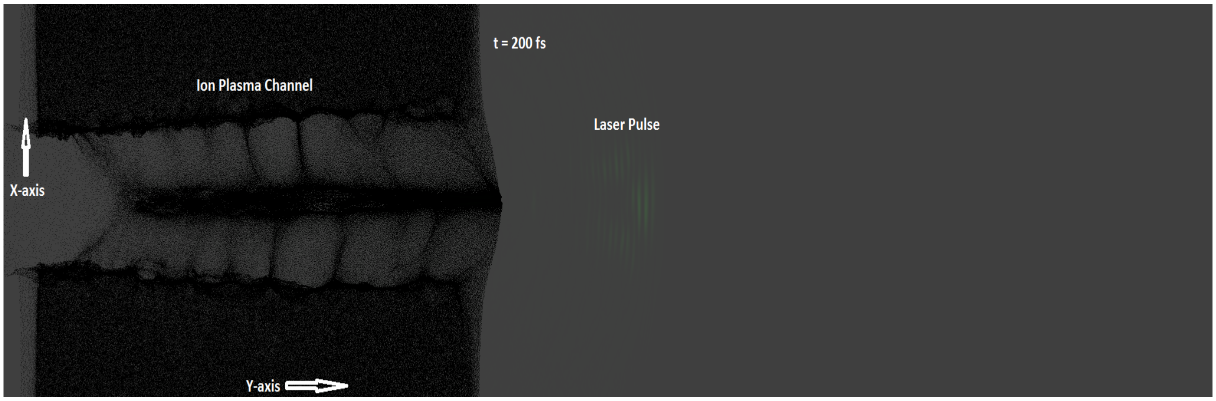

(e)

Figure S2: Evolution of ion density distribution in XY plane at different time instants, (a) 67fs, (b) 100fs, (c) 134fs, (d) 167fs and (e) 200fs. Black colour corresponds to the electron density distribution and green colour is shown for laser pulse.

Figure S1-S2 shows the density distribution of electron and ion at different time instants. The simulation results shown above corresponds to the central slice of NCD plasma target (in XY plane). The results shown above correspond to three dimensional (3D) particle-in-cell (PIC) simulation, where a 2 PW CP laser pulse with a pulse length 20 fs (FWHM) and focal spot diameter of 3 µm (FWHM) is focused on a plasma slab of uniform plasma density and of thickness 30 µm.

It would be of interest to point out here the preference of Gaussian laser beam over flat-top beams (super-Gaussian beams) for MVA ion acceleration. In MVA ion acceleration (which is efficient for thicker target), formation of plasma channel, and generation of axial and azimuthal field depends on density and intensity inhomogeneity. Thus by employing flat top laser pulse of uniform spatial intensity profile (in comparison to Gaussian intensity distribution) it may be difficult to focus it smaller spot size to create self-focused plasma channel and subsequently magnetic field generation, which is important in this mechanism for inducing quasi-static electric field at plasma-vacuum interface. Since flat-top beams evacuate electrons from a larger area from the illuminated part of target accelerated by the radiation pressure, and are more efficient in preventing the radial return of electrons because of higher radial ponderomotive force. Such kind of flat-top beam can be more useful for thin-planar solid target to enhance the efficiency and in reducing the energy spread.
